# Nutritional Imbalances in Polish Children with Coeliac Disease on a Strict Gluten-Free Diet

**DOI:** 10.3390/nu14193969

**Published:** 2022-09-24

**Authors:** Anna Szaflarska-Popławska, Aleksandra Dolińska, Magdalena Kuśmierek

**Affiliations:** 1 Department of Pediatric Endoscopy and Gastrointestinal Function Testing, Ludwik Rydygier Collegium Medicum in Bydgoszcz, Nicolaus Copernicus University in Torun, ul. Jagiellonska 13-15, 85-067 Bydgoszcz, Poland; 2 Department of Pediatrics, Allergology and Gastroenterology, Ludwik Rydygier Collegium Medicum in Bydgoszcz, Nicolaus Copernicus University in Torun, ul. Jagiellonska 13-15, 85-067 Bydgoszcz, Poland

**Keywords:** children, coeliac disease, gluten-free diet, nutritional deficiencies

## Abstract

Currently, the only treatment for coeliac disease (CD) is a strict, lifelong gluten-free diet (GFD); however, their completeness with regard to energy and macro- and micronutrients remains poorly understood. Paediatric studies are often limited by a low quality and a lack of controls, and their findings should be interpreted with caution. The aim of the present study was to evaluate nutritional imbalances in children with CD on a strict GFD. Methods: A single-centre prospective cohort study was conducted. A total of 48 children with CD (33 girls, mean age 11.8 ± 3.68 years) on a strict GFD (mean duration 5.02 ± 3.87 years) were compared with 50 non-coeliac subjects (26 girls, mean age 10.2 ± 3.97 years). In both groups, anthropometric measurements (body height, weight and BMI) and laboratory tests (haemoglobin level, calcium and magnesium serum concentration, folic acid, vitamin B_1_, B_2_, B_6_ and B_12_ level) were checked. Additionally, in coeliac subjects, a 3-day food record for energy and macro- and micronutrient intake assessment were determined, and the values were compared to those in non-CD participants and the dietary reference intake (DRI) standards. Results: The CD children were more likely to demonstrate significantly lower serum vitamin B_1_ and folic acid levels compared to controls (*p* = 0.01 and *p* = 0.002, respectively). Although mean serum calcium values were within normal ranges, they were significantly lower in CD subjects than controls (*p* = 0.01). Mean calcium, folic acid and vitamin D intake was below the dietary recommendations in the CD group (69.9%, 71.2% and 68.9% DRI, respectively) but did not differ significantly between CD and non-coeliac subjects. In turn, the mean supply of proteins and carbohydrates in the CD group substantially exceeded the recommended levels (190.3% and 189.4% DRI, respectively) but was similar to controls. A significantly higher number of CD children were classified as underweight, and a significantly lower number as overweight or obese, compared with controls (*p* < 0.001). Conclusion: Although children with CD receive nutritional education at diagnosis, a GFD often does not provide a balanced set of macro- and micronutrients. This is mainly due to unhealthy dietary habits, as commonly observed in the general population. Children with CD should be informed that while their diet should be free of gluten, it should nevertheless cover all their nutrition requirements in the long term.

## 1. Introduction

Coeliac disease (CD) is an autoimmune enteropathy triggered by gluten in genetically susceptible individuals. The nutritional deficiencies observed in patients with CD may be caused by the disease itself and/or the gluten-free diet (GFD) prescribed in response. The severity of these deficiencies may be affected by the extent and the degree of the small bowel lesions, the time elapsed before diagnosis and after diet administration, as well as the degree of adherence to the GFD [1].

In the last two decades, numerous studies have compared the nutritional quality of gluten-free products with their gluten-containing counterparts [2,3]. The evidence regarding the energy and macro- and micronutrient content of GFDs is controversial. Some research indicates that processed gluten-free products contain less protein and fibre than the gluten-containing equivalents, and higher levels of saturated fat, carbohydrate and salt [4]; however, one study found child-targeted gluten-free products to demonstrate lower levels of sodium, total fat, saturated fat and protein [5]. Some studies indicate that many processed gluten-free products are not as enriched as their gluten-containing counterparts, and therefore may be deficient in some micronutrients, particularly folate, iron, and vitamins B_1_, B_2_ and B_3_ [6]. Despite the differences in nutritional quality between gluten-free products and their gluten-containing counterparts, it is possible to properly balance a GFD by choosing correct products.

A number of studies indicate that children on a GFD do not consume enough nutrient-dense foods to meet all their nutritional recommendations [7,8,9,10,11,12], and that children with CD tend to receive a total protein, fibre, folate, iron, potassium and zinc intakes but high levels of fat, carbohydrate, sugars, FODMAP and sodium. Whether these imbalances reflect a more general trend in the paediatric population or are linked to, or exacerbated by, a GFD remains unclear. Unfortunately, existing data regarding the clinical outcomes of GFD use in children are sparse and inconsistent; even so, some studies indicate low levels of serum folate, vitamin A, D, E, B_12_, magnesium, calcium, iron, zinc, copper and selenium in this group [13,14]. Furthermore, many studies are of low methodological quality and lack controls; as such, their findings should be interpreted tentatively.

Therefore, the aim of the present study was to evaluate haemoglobin level, as well as calcium, magnesium, folic acid and vitamin B_1_, B_2_, B_6_, B_12_ serum levels and dietary intake in children with CD receiving a GFD, and compare these findings with those of non-CD controls receiving a typical diet.

## 2. Methods

The study was performed as a single-centre prospective cohort study and was conducted from 1 January 2016 to 31 July 2019 in the Department of Paediatrics, Allergology and Gastroenterology, Antoni Jurasz University Hospital No. 1 in Bydgoszcz, Poland. The study group comprised 48 subjects (33 girls and 15 boys), aged between 4 and 18 years (mean age 11.8 ± 3.68 years); of these 33 were from the urban environment, who were diagnosed with histologically confirmed CD. The eligibility criteria comprised a duration of GFD ≥ 1 year (mean duration 5.02 ± 3.87 years), strict adherence to a GFD, and absence of co-morbidities. Strict adherence to the GFD was ascertained by evidence of clinical remission, exclusion of gluten transgressions based on detailed dietary interview by the gastroenterologist extensively experienced in the management of CD. Of these, 36 children (75%) were confirmed serologically negative and 2 (4.2%) in histological remission (Marsh 0) in the year preceding the study.

The control group comprised 50 non-coeliac participants hospitalised in the Department of Paediatrics, Allergology and Gastroenterology, including 26 girls and 24 boys aged 4 to 18 years (mean age 10.2 ± 3.97 years), and 33 from the urban environment who declared being on an unrestricted diet. The group included 27 children diagnosed with functional gastrointestinal disorders (all had negative anti-tTG serology) and 23 children without any symptoms suggestive of CD or having an effect on their nutritional intake/status. The inclusion criteria for the control group were: age between 4 and 18 years, absence of organic gastrointestinal disorders, unrestricted diet and normal appetite. The exclusion criteria for the group comprised liver or kidney diseases, acute or chronic inflammation, inflammatory bowel disease, diabetes, chronic asthma and consumption of dietary supplements except for vitamin D. The CD group included a higher proportion of girls (*p* = 0.02) and older children than the control group (*p* = 0.043).

Anthropometric measurements were performed on the two groups. Body weight was measured using a digital scale (calibrated before use, accuracy of 0.1 kg), and body height by a stadiometer. The height and weight measurements were used to calculate BMI (body mass index) (kg/m^2^). The calculations were compared with Polish growth reference charts [15]. BMI-for-age values approximately > +1SD were classified as overweight (equivalent to BMI 25 kg/m^2^ at 18 years), approximately > +2SD as obesity (equivalent to BMI 30 kg/m^2^ at 18 years) and approximately < −1.5SD as underweight (equivalent to BMI 18.5 kg/m^2^ at 18 years).

Laboratory tests, including haemoglobin level, calcium and magnesium serum concentration, folic acid, vitamin B_1_, B_2_, B_6_ and B_12_ level were measured. Haemoglobin concentration was determined by SLS (sodium lauryl sulphate) method (Sysmex Polska Sp. z o.o., Warsaw, Poland). Calcium and magnesium serum concentration were performed by rapid colorimetric method (Siemens Sp. z o.o., Munich, Germany), vitamin B_12_ (Abbott Laboratories Poland Sp. z o.o., Warsaw, Poland) and folic acid serum concentration (Siemens Sp. z o.o., Warsaw, Poland) by chemiluminescence immunoassay; all tests were performed in the Laboratory Diagnostic Division of Jurasz University Hospital in Bydgoszcz and the standards were given by the test manufacturers. Serum vitamin B_1_, B_2_ and B_6_ concentrations were measured using high-performance liquid chromatography (HPLC) in IMD Berlin (Chromsystems Chemicals GMbH, Munich, Germany), the standards were given by the test manufacturer.

Dietary nutritional energy and nutrient intake was assessed using three-day 24 h food recall. Parents and subjects were trained by an experienced clinical dietician to describe precisely all foods consumed during different meals (breakfast, lunch, dinner, snacks) and beverage intake during the three consecutive days, preferably two on weekdays and one at the weekend. They were requested to return the diary as soon as it is completed. The response rate for the dietary recalls in coeliac and non-coeliac groups were 43.8% and 52%, respectively. The completed food diaries were analysed by the same clinical dietician using Dieta 5.D software (Food and Nutrition Board of the Institute of Medicine, Warsaw, Poland, 2011), and compared against the Polish Dietary Reference Values recommended by the National Institute of Public Health-National Institute of Hygiene (NIPH-NIH) in Warsaw, Poland [16]. The results were expressed as percentage of dietary reference intake (%DRI) for total energy, macronutrients (that is, protein, lipids, carbohydrates and fibre) and micronutrient (that is, calcium, magnesium, iron, zinc, folic acid, vitamin A, E, B_1_, B_2_, B_3_, B_6_, C, D, B_12_) intake.

### 2.1. Statistical Analysis

Statistical analysis was performed using STATISTICA13PL. The results for continuous variables are given as mean and standard deviation (SD). The results of non-continuous variables are given as frequency and the percentage. To compare mean values between two groups, Student’s *t*-test was used for normally distributed data or the Mann–Whitney U test for non-normally distributed data. To compare more than two groups, the ANOVA was used for normally distributed data and the Kruskal–Wallis test for non-normally distributed data with appropriate post hoc tests (Scheffé test for ANOVA and Dunn’s test for Kruskal–Wallis test). Statistical differences were considered significant at *p* < 0.05.

### 2.2. Ethical Considerations

The study was approved by the Bioethics Committee of Nicolaus Copernicus University in Toruń (KB 84/2016). Informed consent was obtained from each study participant; the form was signed by a parent/caregiver for children below 16 years, and by both a parent and the child if aged 16 years or older.

## 3. Results

The demographic, clinical and microscopic features of 48 children with histopathologically confirmed CD are summarized in Table 1. The main presenting symptoms at diagnosis were diarrhoea in 27 children (56.3%), abdominal pain in 21 (43.8%) and vomiting in 16 (33.3%). Histopathological findings of duodenal biopsies included complete (37.4%), subtotal (43.8%) or partial (16.7%) villous atrophy.

The anthropometric parameters of CD and control patients are presented in Table 2. In the group of children with CD, all mean values of anthropometric parameters, i.e., weight-for-age z-score, height-for-age z-score and BMI-for-age z-score, were significantly lower than in the control group. A significantly higher number of CD patients were classified as underweight and a significantly lower number as overweight or obese compared with controls (*p* < 0.001).

### 3.1. Haemoglobin and Serum Micronutrient Levels Analysis

Among the vitamins, the most common deficiencies were observed in vitamin B_1_ and folic acid, with mean serum levels of 62.8 ± 12.3 mg/L and 7.9 ± 2.9 ng/mL, respectively; these values were significantly lower than in controls (*p* = 0.01 and *p* = 0.002, respectively). Both deficiencies were observed in seven out of 48 CD children (14.6%). A similar prevalence of serum vitamin B_2_ deficiency was noted in both CD children (8.3%) and controls (8.0%); however, the value was significantly lower in the latter (*p* = 0.001). Mean serum calcium values were within normal ranges but were significantly higher in controls than the CD group (*p* = 0.01); in addition, calcium deficiency was noted in one non-CD participant but none of the CD children.

The mean haemoglobin level and serum values of the selected micronutrients in the CD and control groups are presented in Table 3. The percentages of children with low haemoglobin level and deficient in selected micronutrients in both groups are shown in Table 4.

The differences in mean haemoglobin level and serum values of selected micronutrients in treated CD children were not statistically significant, regardless the duration of GFD (Table 5).

### 3.2. Dietary Intake Assessment

The mean protein and carbohydrate intake in the CD group was well above the dietary recommendations (190.3% and 189.4% DRI, respectively), but no significant difference was observed between the children with CD and non-CD participants. In addition, no difference was found between the two groups in the number of children consuming higher levels of proteins and carbohydrates. Furthermore, no difference in mean energy, lipid or fibre intake was noted between the CD children and controls, and these intakes were in line with the reference values. It was found that 47.6% children with CD consumed less than recommended number of calories, while 42.8% consumed lower amounts of lipids and 33.3% lower amounts of fibre. These percentages were similar between the two groups. The percentage dietary reference intake (%DRI) for total energy and macronutrients are summarized in Table 6, while the numbers of children with normal, low and high %DRI for total energy and macronutrients are presented in Table 7.

In the group of treated children with CD, no statistically significant differences in dietary reference intake (%DRI) for total energy and macronutrients were observed with regard to GFD duration (Table 8).

The mean daily intake of magnesium, zinc, vitamin A, B_1_, B_2_, B_3_, B_6_, B_12_ and C in the children with CD was above dietary recommendations, while the mean calcium, folic acid and vitamin D intakes were low. However, these values did not significantly differ from the mean intake in the control group. In children with CD, the mean daily intake of vitamin E met the nutrient requirements, while in controls, vitamin E intake was significantly lower than in the CD group, and below dietary recommendations (*p* = 0.02) (Table 9).

A high percentage of the CD group demonstrated an insufficient mean daily intake of calcium, magnesium, iron, zinc, folic acid, and vitamins A, E, B_1_, C and D. Their percentages were similar in the two studied groups; however, the vitamin C and E concentrations were significantly higher in controls than in the CD group (*p* = 0.02) (Table 10).

The GFD duration did not have an effect on the mean daily intake of most macro- and micronutrients except for vitamin B_1_. This value was significantly higher in the children receiving a GFD for one to three years than those on the diet for three to six years (Table 11).

## 4. Discussion

Children who have been newly diagnosed with CD often demonstrate nutrient deficiencies [19]. However, it is thought that the majority of subnormal serum/plasma mineral and vitamin levels normalize within 6–12 months after starting a gluten-free diet. Despite this, the gluten-free diet (GFD) itself may be poor in some micronutrients, especially magnesium, zinc, selenium, calcium, vitamin D, vitamin B and folate [20], and it is unknown whether a strict GFD provides a complete energy and macro- and micronutrient intake in children with CD. In addition, little is also known about serum/plasma macro- and micronutritional deficiencies in children with good adherence to GFD.

The present study determined the dietary intake of children with CD by calculating their energy and macro- and micronutrient intake using a three-day 24 h food recall method. All children had been following a GFD for at least one year. These values were compared with age-specific Polish Dietary Reference Values given by the National Institute of Public Health-National Institute of Hygiene in Warsaw, Poland, and with the dietary intake of non-coeliac children on a non-gluten-free diet. It was found that both study groups followed an unbalanced diet characterized by a higher than recommended consumption of proteins and carbohydrates. Moreover, neither group of participants complied with the recommendations for optimal intake with regard to most studied minerals (calcium, magnesium, zinc) and vitamins (folic acid, vitamin A, B_1_, B_2_, B_3_, B_6_, C, B_2_, D_3_). In addition, no correlation was found between the clinical form of CD, the degree of villous atrophy at the diagnosis, the area of residence and the nutrient deficiencies for all measured nutrients (data not shown).

Several studies indicate that children with CD demonstrate higher total carbohydrate intakes than given in recommendations [7,8]. Indeed, in our cohort of children with CD on a strict GFD, more than 95% had a higher carbohydrate intake, with the mean daily intake being nearly 90% higher than recommended. This is not surprising, as gluten-free products are often supplemented with carbohydrates to improve their texture and palatability [8] and present a high glycaemic index (GI) [21,22]. However, as also noted previously [7,8,9], our findings do not indicate any significant difference in carbohydrate intake between children on gluten-free and gluten-containing diets, suggesting that this imbalance is not exacerbated by GFD. The higher intake may well be attributable to the Polish population, particularly schoolchildren and young adults, following Western dietary habits, which includes foods with high simple sugar levels (e.g., sugar sweetened beverages) [23,24]. Although it was not possible to calculate the GI of the GFD in the CD-affected children in the present study, due to the limited availability of information, it is well known that gluten slows the rate of small bowel starch hydrolysis in the small bowel; as such, dietary gluten elimination may well augment the glycaemic response to carbohydrates. Therefore, when combined with a high carbohydrate intake, such elimination may impair glycaemic control in children with CD and coexistent type 1 diabetes and increase the risk of overweight and obesity, dyslipidaemia, cardiovascular and liver disease in the general population [25]. It has been reported that the risk of paediatric obesity and metabolic syndrome can also be further increased by excessive protein intake [10,23]; indeed, our present findings indicate no significant differences between the GFD and control groups. It is therefore recommended that the children should receive dietary counselling to limit carbohydrate and protein intake.

Unexpectedly, our findings indicate that both groups of children consumed a relatively good amount of fibre. Existing literature regarding fibre intake in children with CD is rather conflicting, with some of them indicating sufficient intake in CD patients [7,10] and others low intake [9,11,23,26]. Reports indicate that gluten-free products are often composed of low-fibre refined flours and starches [11], suggesting that children on a GFD may be at a greater risk of low fibre intake than those following a broader diet. However, the range of gluten-free high-fibre grains have gradually expanded within the last decade, and this could account for the recently observed increase in consumption of dietary fibre by children on gluten-exclusion diets.

The anaemia found in CD subjects has a multifactorial pathogenesis. The most common factor is iron deficiency; however, malabsorption of folate and vitamin B_12_, a blood loss and chronic anaemia may also play a role [27]. The prevalence of iron deficiency anaemia was estimated at between 4.3 at 24.9% in children with newly diagnosed CD in 5 countries in Central Europe [28], which is a figure close to ours. A normalisation of the haemoglobin level was demonstrated after about 6–12 months from the beginning of the GFD alone, following the recovery of the integrity of the small intestinal mucosa [29]. In our group of paediatric patients receiving at least one year of strict GFD, three patients remained anaemic. However, the mean haemoglobin level was similar between coeliac and non-coeliac subjects, as was the percentage of children with low haemoglobin level. It seems that a strict GFD alone is sufficient to maintain a normal haemoglobin level as our anaemic children are usually not prescribed oral iron supplementation. In addition, it can be hypothesized that an iron deficiency anaemia in Polish children may be associated with diet low in iron regardless of whether it contains gluten.

As noted previously [7,8,9], the children with CD on GFD demonstrated a lower intake of some minerals and vitamins than recommended. Most patients failed to meet the Recommended Dietary Allowances of vitamin D, folic acid and calcium; however, it was usually not related to the GFD, because it also applied to patients not on an elimination diet.

Vitamin D deficiency may be of special importance, since children with CD have been found to suffer a higher prevalence of low bone mineral density [30]. However, vitamin D deficiency has also been noted in the general population [31], with a prevalence as high as 84.2% in healthy Polish preadolescents after winter [32]. This is highly surprising because, according to Polish guidelines [31], vitamin D should be supplemented from at least September to May, or in the case of insufficient insolation, throughout the year on a daily basis. It is possible that the vitamin D deficiency observed in Polish population could at least partially be due to insufficient intake, being observed in nearly all study participants. However, it should be emphasized that a diet, even one rich in nutritional sources of vitamin D_3_, can only cover a maximum of 10% of the daily requirement. Optimal vitamin D_3_ concentration is obtained primarily from skin synthesis and adequate supplementation [32]. Of note, in the present study, only a few children (a total of 6 coeliac and 7 non-coeliac subjects) were supplemented with vitamin D_3_ and the schedule of its administration was not uniform. Due to expected difficulty in interpreting the results, no analysis of serum vitamin D_3_ concentration was performed in the present study. No significant differences in mean daily intake or percentage of patients with low vitamin D consumption were found between the two studied groups. Hence, our observations likely reflect an imbalance in vitamin D intake in the general population, unrelated to the use of a GFD. There is no evidence suggesting that vitamin D supplementation offers any advantage in CD patients over following a GFD alone.

Another micronutrient playing a vital role in bone metabolism is calcium. Calcium intake in CD patients varies considerably between studies and this may be due to differences in regional consumption and the availability of calcium-dense food products. The proportion of children with CD with inadequate calcium intake in our study was 61.9%, with no significant difference noted between CD children and controls, suggesting that Polish children are at risk of insufficient calcium intake, regardless of whether they are on a GFD. A similar value was reported by Alzaben et al. [8]. By contrast, some authors found treated CD patients to demonstrate 100% recommended calcium consumption [33]. It should be noted, however, that despite the inadequate calcium intake, none of the examined children with CD demonstrated decreased serum calcium concentration; this may suggest the activation of a compensatory mechanism, i.e., an increase in the parathyroid hormone concentration, which improves the absorption of calcium and vitamin D_3_ [34]. As it is well known that patients with CD are at risk of suboptimal bone health [30], calcium intake recommendations for CD patients should be adjusted by national scientific organisations.

Only a few studies have investigated folate intake in CD children adhering to a GFD. Some report that CD individuals consumed the recommended amount of folate [33], whereas others indicate deficiency [9,26,35]. In our study, both CD and non-CD children were below the recommended folate intake level, with no significant difference noted between the two groups. However, the mean serum folate level was significantly lower in the children with CD than in their non-CD peers, and more children on GFD were folate deficient (this difference was not statistically significant). This is not surprising, because the mandatory folate fortification regulations for cereal products do not apply to their gluten-free counterparts. It has been suggested that in coeliac patients following a GFD, serum folate level should be regularly measured and supplemented in subjects with folate deficiency [12]. Of note, some gluten-free pseudocereals, e.g., quinoa and amaranth contain more folic acid than wheat; therefore, their incorporation into the gluten-free diet should be promoted [36].

Regarding B group vitamins (other than folate), in the present study, the mean daily intake of vitamins B_1_, B_2_, B_3_, B_6_ and B_12_ did not differ significantly between the CD and control groups, both of whose intakes exceeded the daily requirement. In both groups of children, the mean serum vitamin B_1_, B_2_ and B_12_ concentration (no vitamin B_3_ tests were performed) was within the normal range. While the concentration of vitamin B_6_ was above the upper limit of the normal range, no statistically significant differences were found between the groups. The obtained data indicate that the observed abnormalities do not result from a gluten exclusion diet, but from the inappropriately composed diet consumed by Polish children as a whole. Our data is partly in line with those from other countries, which are often inconsistent. Salazar Quero et al. [37] report that vitamin B_6_ and B_12_ intake in children with CD significantly exceeded local recommendations. A Swedish study found significantly higher vitamin B_1_, B_6_ and B_12_ consumption in patients with CD than in controls [35]; however, a Canadian study found no such differences [8], and Turkish and Spanish studies found lower B vitamin consumption in the study group than the controls [9,26]. These differences probably result from a combination of country-specific dietary habits and nutritional preferences, and the different composition of local gluten-free products; therefore, it seems that nutritional recommendations for patients with CD should be created locally.

Data on the effects of GFD on the anthropometric parameters of patients with CD are conflicting. Some authors indicate that good compliance to a GFD is associated with normalization of BMI in both underweight and overweight children and an acceleration of linear growth [38,39]. Other studies report that gluten-free diets have a negative effect on anthropometric parameters, with an increased prevalence of overweight and obesity [40]. Moreover, it has been reported that in children with initially low coeliac-related body weight, a complete restoration of body weight is achieved after 6–12 months of GFD [41]. In the present study, the children following a GFD for at least one year demonstrated significantly lower BMI when compared to non-coeliac children, and 11 children with low body weight at diagnosis remained underweight. It may be hypothesized that other factors must contribute to poor weight gain in our CD groups, since poor compliance to a GFD was excluded. It is recommended that paediatricians closely check the anthropometric parameters of a child diagnosed with coeliac disease, regardless of adherence to the diet.

One limitation of the study is the relatively small sample size that was dietetically assessed. In addition, the control group comprised non-coeliac children who were not devoid of some health problems. However, they were elaborately selected, and the symptoms and conditions were assessed as not having an effect on their nutritional intake/nutritional status. Secondly, the use of three-day recalls for dietary intake assessment is a retrospective method; this can favour both under- and overreporting, especially in children and adolescents who may not be concerned about correctly reporting meal patterning (i.e., food size and proportions). However, some data indicate that 3-day recall has a reasonable validity for estimating nutrient intake [42]. In addition, since dietary composition varies between regions, our findings cannot be generalized to individuals other than the studied age groups and from other geographical locations. Finally, in Poland, as in other countries [6], little data is available on the micronutrient profile of gluten-free products, and hence our estimates of the mean micronutrient consumption in our CD group may not be completely accurate.

Nevertheless, the study does have several strong points. Firstly, in addition to the dietary nutrient intake in children and adolescents with CD, the study also includes the serum levels of some minerals and vitamins. Secondly, unlike previous studies, the obtained data is compared with both national nutritional recommendations and a non-CD control group. Finally, the studied group was meticulously checked by an experienced dietician for strict adherence to the GFD, and even a small dietary transgression resulted in exclusion from the study group.

## 5. Conclusions

The study shows that children with CD strictly adhering to a gluten-free diet are at risk of consuming excessive amounts of carbohydrates and proteins and insufficient amounts of, inter alia, calcium, folic acid and vitamin D. However, these imbalances do not seem to be exacerbated by a gluten-free diet, regardless of its duration. Every effort should be undertaken to incorporate regular dietary counselling into the therapeutic follow-up of CD individuals. Dietary education based on GFDs should not only teach the children and their guardians how to avoid gluten, but also how to comply with national dietary recommendations.

## Figures and Tables

**Table 1:** Main general characteristics of the coeliac disease subjects on a strict gluten-free diet.

	Coeliac Disease Subjects (*n* = 48)
Age in years, mean ± SD	11.8 ± 3.68
Girls, *n* (%)	33 (68.7)
BMI z-score, mean ± SD	−0.01 ± 1.19
Main presenting symptoms at diagnosis, *n* (%)	
Diarrhoea	27 (56.3)
Vomiting	16 (33.3)
Flatulence	12 (25.0)
Abdominal pain	21 (43.8)
Leg pain	1 (2.1)
Skin lesions	1 (2.1)
Constipation	7 (14.6)
Hair loss	1 (2.1)
Short stature and/or failure to thrive	17 (35.4)
Anaemia	8 (16.7)
No symptoms	7 (14.6)
Clinical form of the disease [17], *n* (%)	
classical	25 (52.1)
non-classical	16 (33.3)
asymptomatic	7 (14.6)
Marsh’s classification at diagnosis [18], *n* (%)	
2	1 (2.1)
3a	8 (16.7)
3b	21 (43.8)
3c	15 (31.3)
4	3 (6.1)
Duration of gluten-free diet in years, mean ± SD	5.02 ± 3.87
1–3 years, *n* (%)	15 (31.3)
3–6 years, *n* (%)	23 (47.9)
>6 years, *n* (%)	10 (20.8)

**Table 2:** Anthropometric parameters of coeliac disease patients and control subjects compared with weight, height and body mass index (BMI) standards for Polish children [15].

Parameter		Coeliac Disease Subjects *n* = 48	Control Subjects *n* = 50	*p*
Weight-for-age Z-score Mean (SD)		−0.58 (1.28)	0.42 (0.98)	0.0000
Height-for-age Z-score Mean (SD)		−0.06 (1.17)	0.57 (1.15)	0.0093
BMI-for-age Z-score Mean (SD)		−0.01 (1.19)	−0.57 (1.39)	0.0345
*n* (%) of subjects	Underweight	16 (33.3%)	3 (6.0%)	<0.001
	Overweight	3 (6.3%)	9 (18.0%)	
	Obesity	0 (0%)	1 (2.0%)	

Overweight-approximately > +1SD (equivalent to BMI 25 kg/m^2^ at 18 years), obesity-approximately > +2SD (equivalent to BMI 30 kg/m^2^ at 18 years), underweight-approximately < −1.5SD (equivalent to BMI 18.5 kg/m^2^ at 18 years).

**Table 3:** Mean haemoglobin level and serum values of the selected micronutrients in CD and non-CD participants.

	Normal Values	Coeliac Subjects *n* = 48 Mean (SD)	Control Subjects *n* = 50 Mean (SD)	*p*
Vitamin B_1_ (mg/L)	>49	62.8 (12.3)	69.0 (11.3)	0.01
Vitamin B_2_ (mg/L)	180–295	254.7 (47.7)	226.8 (33.4)	0.001
Vitamin B_6_ (mg/L)	8.7–27.2	30.0 (8.1)	29.9 (10.7)	0.92
Vitamin B_12_ (pg/mL)	179–1162	415.3 (157.6)	479.7 (246.4)	0.13
Folic acid (ng/mL)	4.8–19	7.9 (2.9)	10.2 (4.3)	0.002
Calcium (mmol/L)	2.2–2.7	2.4 (0.1)	2.5 (0.1)	0.01
Magnesium (mmol/L)	0.66–0.87	0.9 (0.1)	0.8 (0.1)	0.1
Haemoglobin (g/dL)	4–6 years 11.5–14.5	13.1 (0.5)	12.3 (1.1)	0.39
	6–16 years 12.0–15.0	13.4 (1.0)	13.3 (1.0)	0.8
	Girls > 16 years 11.2–15.7	12.5 (0.6)	12.7 (1.1)	0.7
	Boys > 16 years 13.7–17.5	14.9 (1.2)	Not applicable	

**Table 4:** Number of CD and control subjects with micronutrient deficiencies.

	Number (%) of Coeliac Subjects with Deficiency *n* = 48	Number (%) of Control Subjects with Deficiency *n* = 50	*p*
Vitamin B_1_	7 (14.6)	1 (2.0)	0.023
Vitamin B_2_	4 (8.3)	4 (8.0)	0.95
Vitamin B_6_	0 (0)	0 (0)	0.99
Vitamin B_12_	1 (2.1)	1 (2.0)	0.99
Folic acid	7 (14.6)	4 (8.0)	0.30
Calcium	0 (0)	1 (2.0)	0.99
Magnesium	0 (0)	0 (0)	0.99
Haemoglobin	3 (6.2)	3 (6.0)	0.85

**Table 5:** Mean haemoglobin level and serum values of selected micronutrients in CD children with regard to GFD duration.

	Duration of Gluten-Free Diet			
	1–3 Years *n* = 15 Mean (SD)	3–6 Years *n* = 23 Mean (SD)	>6 Years *n* = 10 Mean (SD)	*p*
Vitamin B_1_ (mg/L)	58.9 (11.2)	63.2 (13.2)	67.6 (10.5)	0.21
Vitamin B_2_ (mg/L)	267.1 (44.4)	255.0 (52.2)	235.3 (38.1)	0.27
Vitamin B_6_ (mg/L)	29.6 (7.3)	31.6 (8.8)	27.0 (7.4)	0.32
Vitamin B_12_ (pg/mL)	401.3 (130.3)	435.4 (163.3)	390.2 (189.8)	0.7
Folic acid (ng/mL)	7.6 (3.0)	8.7 (2.9)	6.4 (2.1)	0.11
Calcium (mmol/L)	2.4 (0.1)	2.4 (0.1)	2.4 (0.1)	0.5
Magnesium (mmol/L)	0.9 (0.1)	0.9 (0.1)	0.9 (0.0)	0.98
Haemoglobin (g/dL)	13.1 (1.2)	13.5 (1.0)	13.7 (0.3)	0.3

**Table 6:** Mean daily intake and percentage of dietary reference intake (%DRI) for total energy and macronutrients in CD and control subjects.

Variable	Coeliac Group *n* = 21		Control Group *n* = 26		*p*
	Mean (SD)	%DRI Mean (SD)	Mean (SD)	%DRI Mean (SD)	
Energy, kcal/day	1845.2 (737.9)	90.0 (13.2)	1755.5 (273.0)	91.9 (17.2)	0.68
Proteins, g/day	81.7 (22.1)	190.3 (89.4)	78.1 (22.9)	213.0 (88.0)	0.39
Lipids, g/day	63.3 (11.5)	97.4 (24.5)	56.5 (13.9)	91.5 (23.8)	0.41
Saturated fat, g/day	28.7 (4.7)	140.0 (30.2)	25.6 (6.1)	131.3 (31.4)	0.35
Monounsaturated fat, g/day	23.2 (5.6)	56.5 (14.1)	20.7 (6.7)	53.1 (12.4)	0.38
Polyunsaturated fat, g/day	6.6 (1.9)	36.7 (12.3)	5.8 (1.6)	33.1 (10.35)	0.28
Carbohydrates, g/day	255.8 (26.2)	189.4 (35.8)	250.4 (46.8)	192.6 (36.8)	0.77
Fibre, g/day	21.1 (5.6)	115.9 (35.7)	19.2 (5.7)	109.4 (29.3)	0.5

**Table 7:** Numbers of children with normal, low and high dietary reference intake (%DRI) for total energy and macronutrients in CD and control subjects.

%DRI	Coeliac Group *n* = 21 Number (%)			Control Group *n* = 26 Number (%)			*p*
	Normal	Low	High	Normal	Low	High	
Energy	10 (47.6)	10 (47.6)	1 (4.8)	13 (50.0)	11 (42.3)	2 (7.7)	0.88
Proteins	3 (14.3)	1 (4.8)	17 (80.9)	2 (7.7)	0 (0)	24 (92.3)	0.32
Lipids	6 (28.6)	9 (42.8)	6 (28.6)	7 (26.9)	13 (50.0)	6 (23.1)	0.87
Carbohydrates	0 (0)	1 (4.8)	20 (95.2)	0 (0)	1 (3.8)	25 (96.2)	0.87
Fibre	3 (14.3)	7 (33.3)	11 (52.4)	6 (23.1)	8 (30.8)	12 (46.1)	0.74

**Table 8:** Percentage of dietary reference intake (%DRI) for total energy and macronutrients in coeliac patients with regard to the duration of the gluten-free diet.

	Duration of Gluten-Free Diet			
	1–3 Years *n* = 7 Mean (SD)	3–6 Years *n* = 11 Mean (SD)	>6 Years *n* = 3 Mean (SD)	*p*
Energy	93.7 (11.5)	87.2 (15.6)	91.7 (4.0)	0.6
Proteins	223.9 (114.5)	167.0 (79.7)	197.3 (42.7)	0.44
Lipids	101.0 (20.6)	95.4 (28.1)	96.7 (27.0)	0.9
Carbohydrates	203.4 (17.0)	179.9 (44.9)	191.3 (26.8)	0.42
Fibre	134.4 (47.7)	106.6 (25.9)	107.0 (28.4)	0.25

**Table 9:** Mean daily intake and percentage of dietary reference intake (%DRI) for micronutrients in CD and control subjects.

Variable	Coeliac Group *n* = 21		Control Group *n* = 26		*p*
	Mean (SD)	%DRI Mean (SD)	Mean (SD)	%DRI Mean (SD)	
Calcium, mg/day	817.4 (291.9)	69.9 (29.2)	673.6 (297.9)	59.0 (29.3)	0.21
Magnesium, mg/day	292.8 (78.6)	133.6 (77.6)	275.9 (74.7)	135.4 (65.0)	0.93
Iron, mg/day	12.1 (2.5)	101.2 (26.6)	11.5 (4.0)	105.9 (43.9)	0.68
Zinc, mg/day	9.6 (2.1)	130.9 (57.8)	8.9 (2.3)	128.2 (40.3)	0.85
Folic acid, μg/day	239.8 (46.7)	71.2 (18.6)	222.4 (52.2)	74.4 (25.5)	0.65
Vitamin A, μg/day	981.8 (452.9)	160.8 (91.0)	751.6 (410.2)	122.6 (57.5)	0.09
Vitamin E, mg/day	7.1 (2.6)	97.8 (39.6)	5.8 (2.0)	75.0 (26.3)	0.02
Vitamin B_1,_ mg/day	1.3 (0.4)	117.8 (37.9)	1.2 (0.4)	127.8 (43.0)	0.41
Vitamin B_2,_ mg/day	1.8 (0.4)	165.4 (48.6)	1.6 (0.4)	178.1 (59.7)	0.43
Vitamin B_3_, mg/day	23.8 (12.6)	170.6 (100.5)	21.6 (13.0)	179.9 (119.5)	0.78
Vitamin B_6,_ mg/day	2.1 (0.5)	169.2 (57.0)	1.8 (0.6)	179.4 (74.5)	0.61
Vitamin C, mg/day	75.9 (38.4)	130.0 (69.6)	70.7 (39.4)	129.0 (81.3)	0.97
Vitamin B_12,_ μg/day	3.1 (0.5)	143.7 (41.8)	3.2 (1.1)	177.4 (88.0)	0.11
Vitamin D, μg/day	1.6 (0.7)	68.9 (263.6)	1.7 (0.8)	11.3 (5.5)	0.27

**Table 10:** Number of children with normal, low and high dietary reference intake (%DRI) for micronutrients in coeliac and control subjects.

%DRI	Coeliac Group *n* = 21 Number (%)			Control Group *n* = 26 Number (%)			*p*
	Normal	Low	High	Normal	Low	High	
Calcium	6 (28.6)	13 (61.9)	2 (9.5)	3 (11.5)	22 (84.7)	1 (3.8)	0.2
Magnesium	1 (4.8)	10 (47.6)	10 (47.6)	1 (3.8)	10 (38.0)	15 (58.0)	0.78
Iron	9 (42.8)	6 (28.6)	6 (28.6)	6 (23.1)	11 (42.3)	9 (34.6)	0.33
Zinc	2 (9.5)	7 (33.3)	12 (57.2)	1 (3.8)	8 (30.8)	17 (65.4)	0.69
Folic acid	3 (14.3)	18 (85.7)	0 (0)	4 (15.3)	20 (77.0)	2 (7.7)	0.28
Vitamin A	1 (4.8)	5 (23.8)	15 (71.4)	2 (7.7)	9 (34.6)	15 (57.7)	0.61
Vitamin E	5 (23.9)	9 (42.8)	7 (33.3)	3 (11.5)	20 (77.0)	3 (11.5)	0.02
Vitamin B_1_	9 (42.8)	4 (19.1)	8 (38.1)	7 (26.9)	5 (19.2)	14 (53.9)	0.47
Vitamin B_2_	1 (4.8)	1 (4.8)	19 (90.4)	0 (0)	2 (7.7)	24 (92.3)	0.41
Vitamin B_3_	5 (23.8)	3 (14.3)	13 (61.9)	6 (23.1)	4 (15.4)	16 (61.5)	0.99
Vitamin B_6_	1 (4.8)	1 (4.8)	19 (90.4)	1 (3.8)	2 (7.7)	23 (88.5)	0.91
Vitamin C	4 (19.1)	7 (33.3)	10 (47.6)	0 (0)	12 (46.1)	14 (53.9)	0.02
Vitamin B_12_	1 (4.8)	1 (4.8)	19 (90.4)	3 (11.5)	2 (7.7)	21 (80.0)	0.62
Vitamin D	0 (0)	20 (95.2)	1 (4.8)	0 (0)	26(100.0)	0 (0)	0.2

**Table 11:** Percentage of dietary reference intake (%DRI) for micronutrients in CD patients with regard to gluten-free diet duration.

	Duration of Gluten-Free Diet			
	1–3 Years *n* = 7 Mean (SD)	3–6 Years *n* = 11 Mean (SD)	>6 Years *n* = 3 Mean (SD)	*p*
Calcium	65.7 (33.9)	79.7 (24.7)	43.7 (20.2)	0.15
Magnesium	158.6 (101.1)	125.7 (71.4)	104.3 (15.0)	0.56
Iron	111.6 (29.2)	99.0 (26.1)	85.3 (18.5)	0.35
Zinc	158.7 (78.2)	113.2 (44.2)	131.0 (30.1)	0.28
Folic acid	73.4 (22.6)	72.4 (18.4)	62.3 (3.5)	0.69
Vitamin A	140.7 (81.4)	172.9 (103.5)	163.3 (84.6)	0.78
Vitamin E	99.6 (45.6)	105.4 (37.3)	65.7 (24.0)	0.32
Vitamin B_1_	142.7 (39.4)	99.1 (29.2)	128.0 (34.7)	0.04
Vitamin B_2_	171.0 (39.8)	166.2 (58.5)	149.3 (34.4)	0.82
Vitamin B_3_	209.6 (127.0)	133.4 (54.6)	216.0 (146.9)	0.21
Vitamin B_6_	190.7 (60.2)	152.3 (51.6)	181.0 (70.1)	0.37
Vitamin C	144.4 (87.2)	127.6 (63.0)	104.7 (63.1)	0.72
Vitamin B_12_	141.1 (28.7)	140.6 (47.3)	161.0 (57.7)	0.76
Vitamin D	9.29 (3.25)	121.6 (364.0)	15.0 (8.0)	0.65

## Data Availability

The data presented in this study are available on request from the corresponding author.
